# Tough Way In, Tough Way Out: The Complex Interplay of Host and Viral Factors in Nucleocytoplasmic Trafficking during HIV-1 Infection

**DOI:** 10.3390/v14112503

**Published:** 2022-11-12

**Authors:** Satarupa Sarkar, Kannan Balakrishnan, Kumaraswami Chintala, Krishnaveni Mohareer, Tom Luedde, Ananda Ayyappan Jaguva Vasudevan, Carsten Münk, Sharmistha Banerjee

**Affiliations:** 1 Department of Biochemistry, School of Life Sciences, University of Hyderabad, Gachibowli, Hyderabad 500046, India; 2 Clinic for Gastroenterology, Hepatology, and Infectiology, Medical Faculty, Heinrich Heine University Düsseldorf, 40225 Düsseldorf, Germany; 3 Florida Research and Innovation Center, Cleveland Clinic, Port St. Lucie, FL 34987, USA; 4 Structural Cell Biology Group, Genome Integrity, and Structural Biology Laboratory, National Institute of Environmental Health Sciences (NIEHS), National Institute of Health, Research Triangle Park, Durham, NC 27709, USA

**Keywords:** HIV-1, nucleocytoplasmic trafficking, capsid import, viral mRNA export, HIV-1 Rev, nucleoporins

## Abstract

Human immunodeficiency virus-1 (HIV-1) is a retrovirus that integrates its reverse-transcribed genome as proviral DNA into the host genome to establish a successful infection. The viral genome integration requires safeguarding the subviral complexes, reverse transcription complex (RTC) and preintegration complex (PIC), in the cytosol from degradation, presumably effectively secured by the capsid surrounding these complexes. An intact capsid, however, is a large structure, which raises concerns about its translocation from cytoplasm to nucleus crossing the nuclear membrane, guarded by complex nuclear pore structures, which do not allow non-specific transport of large molecules. In addition, the generation of new virions requires the export of incompletely processed viral RNA from the nucleus to the cytoplasm, an event conventionally not permitted through mammalian nuclear membranes. HIV-1 has evolved multiple mechanisms involving redundant host pathways by liaison with the cell’s nucleocytoplasmic trafficking system, failure of which would lead to the collapse of the infection cycle. This review aims to assemble the current developments in temporal and spatial events governing nucleocytoplasmic transport of HIV-1 factors. Discoveries are anticipated to serve as the foundation for devising host-directed therapies involving selective abolishment of the critical interactomes between viral proteins and their host equivalents.

## 1. Introduction

Viruses carry DNA or RNA as their genomic blueprints for propagating inside prokaryotic or eukaryotic hosts. In eukaryotes, most DNA viruses, and a few RNA viruses, have to complete the daunting task of crossing the nuclear membrane twice; first, to enter the nucleus to replicate their genomes, and second to export their RNA and other factors out of the nucleus for translation of viral proteins and their assembly to form new progeny. Viruses have evolved adequate mechanisms and bear factors that aid in hijacking host import–export machinery across the nuclear membrane and completing the productive infection. While there may be overlapping host players and common mechanisms employed in infections caused by different viruses, the strategies employed by a specific virus always involve certain unique steps. DNA viruses, such as baculoviruses, herpes viruses, or adenoviruses, dock at the nuclear pore complex (NPC), but not all viruses pass through NPC to enter the nucleus with their intact capsids. Some other DNA viruses, such as Hepatitis B viruses, disassemble their capsids once they have crossed the nuclear membrane at the nucleoplasm, while others, such as parvoviruses and human papillomaviruses, employ NPC-independent mechanisms. Genomes of most RNA viruses are not required to enter the nucleus, but retroviruses and orthomyxoviruses deliver their genomes into the nucleus and replicate there, again, using their unique mechanisms. Retroviruses, such as human immunodeficiency virus, type-1 (HIV-1), are unique in integrating their reverse-transcribed DNA into the host genome (also called proviral DNA). With the incorporation of proviral DNA, retroviruses have also evolved tactics to facilitate the nuclear export of viral factors, including viral RNA and proteins, to the cytoplasm, without which the infection cycle remains incomplete.

The focus of this review is to present the current developments in the nucleocytoplasmic shuttling of RNAs and proteins of HIV-1, whose infection remains a major global health concern with no cure or vaccine. Current anti-retroviral therapy (ART) includes a combination of drugs targeting different stages of the HIV-1 lifecycle, such as entry into human cells, reverse-transcription of its RNA genome, integration of proviral DNA into the host DNA, proteolytic cleavage of viral polyproteins and budding of new virions. Although nucleocytoplasmic transport of viral factors has been investigated as a potential target, currently, there is no successful antiviral agent that blocks nucleocytoplasmic transport of viral factors. The review covers a brief overview of the host nuclear transport machinery and discusses the recent advances in nucleocytoplasmic transport of HIV factors across the nuclear membrane. It tabulates an expanding list of associated host factors, tracking the temporal and spatial events of the lifecycle of HIV-1 that require nucleocytoplasmic shuttling, and deliberates on elusive events, such as, which side of the nuclear membrane, the capsid dissemination and reverse transcription begins and culminates, and points to pertinent questions and un-investigated areas in the concluding remarks.

### The Cellular Nuclear Transport Machinery

Physiological macromolecule movement across the nuclear membrane is highly regulated at multiple levels, including permeability barriers erected by NPCs, nuclear localization signal (NLS), and (or) nuclear export signal (NES) on the molecules being transported, and their cognate soluble import and export receptors. The nuclear transport machinery consists of NPCs embedded in the nuclear envelope, which fuse the outer and inner nuclear membrane to form the channel, with several associated transport and accessory factors on either side of the nuclear membrane. Nuclear pores have annulated scaffolds built from several copies of about 30 different nucleoporin (NUP) proteins, mimicking tubes connecting either side of the nuclear membrane. Fibrous appendages protrude from the cytoplasmic and nucleoplasmic surfaces of the NPC. These appendages from the nucleoplasmic surface of NPC form a distinctive structure called the “nuclear basket” in the nucleus [1]. Into the central channel protrudes a dense meshwork of intrinsically disordered chains of NUPs rich in phenylalanine (F) and glycine (G) repeats, termed FG-NUPs. It is these FG-NUPs in the NPC that create the permeability barrier across the nuclear membrane, through which small molecules less than 40 kDa diffuse freely; however, the movements of macromolecules greater than 40 kDa are impaired [2,3]. Several models, including the “selective phase” model [4], the “brush” model [5], and the “virtual gate” model [6], have been proposed to define the mechanism of the functionality of the FG-NUPs-based permeability barrier. For the structural and functional details about NPC, please refer to the reviews [7,8] and the references therein. Briefly, the transit of macromolecules (>40 kDa) through NPCs is supported by nuclear transport proteins/receptors (also referred to as importins and exportins of the karyopherin family) that bind to their respective cargoes, move through the NPC channel, and release the cargo on the other side [9,10]. Macromolecules including histones, transcription factors, ribonucleoproteins (RNPs), and RNAs take advantage of the ability of nuclear transport receptors to traffic between the nucleus and cytosol. The energy required for this transport across the nuclear membrane appears to be more for binding and release of cargo than for the movement through the channels and may exemplify a thermal ratchet [10]. Classical examples of such active carrier proteins are importin-α and CRM1 that recognize NLS and NES in their cargoes, respectively. For instance, the CRM1-RanGTP complex binds to its cargo in the nucleus, which is released into the cytoplasm upon GTP hydrolysis, facilitated by the GTPase-activating protein, RanGAP, in the cytoplasm [11,12]. Similarly, nuclear RNA export factor 1 (NXF1) that heterodimerizes with NTF2-related export protein-1 (NXT1) is involved in the export of fully processed mRNA from the nucleus to the cytoplasm [13]. The binding and release of mRNA cargo by the NXF1:NXT1 carrier are coordinated by RNA structure alteration by ATP-powered DEAD-box helicases [13]. Thus, NPCs and a number of accessory transport factors or carrier proteins that cycle between the nuclear and cytoplasmic compartments make an efficient nucleocytoplasmic trafficking machinery. A comprehensive overview of the cellular nuclear transport machinery can be obtained in an earlier review [14] and references therein.

## 2. The Interactions of HIV-1 with the Cellular Nuclear Transport Machinery

HIV-1 is an enveloped virion that houses a conical core made up of viral capsid protein (CA), which encloses two copies of positive-sense single-stranded RNA molecules, viral reverse transcriptase (RT), viral integrase (IN), viral protease (PR), and viral protein R (Vpr). The viral RNA is tightly bound to the nucleocapsid protein (p7). The viral membrane originates from the plasma membrane of the infected host cell but is embedded with viral envelope protein Env, a heterodimer of gp120 and gp41. Membrane fusion is the key mechanism of entry into a host cell by HIV-1 carried out by Env, which makes contact with the primary host cell surface receptor CD4 and the coreceptors, CCR5 or CXCR4 [15]. The interactions are accompanied by large structural rearrangements in viral Env protein, chiefly gp41 that induces viral and host membrane fusion allowing viral entry (for detailed reviews on the molecular mechanism of HIV-1 entry and its cell tropism, please refer to [15,16] and references therein). Subsequent to membrane fusion, the viral capsid is released into the host cell cytoplasm.

### 2.1. HIV-1’s Journey from the Cytoplasm to the Nucleus

Although the events occurring between the entry into the host cell and integration into the host genome during HIV-1 infection are not completely clear, the transportation of viral capsid to the cytoplasmic side of the nuclear envelope marks the beginning of the interplay of HIV-1 with cellular nuclear trafficking machinery. The section below discusses the recent advances that elucidate these early events, focusing on the crosstalk of viral components with nuclear trafficking machinery.

#### 2.1.1. Nuclear Entry of HIV-1 Capsid

**Figure 1:**
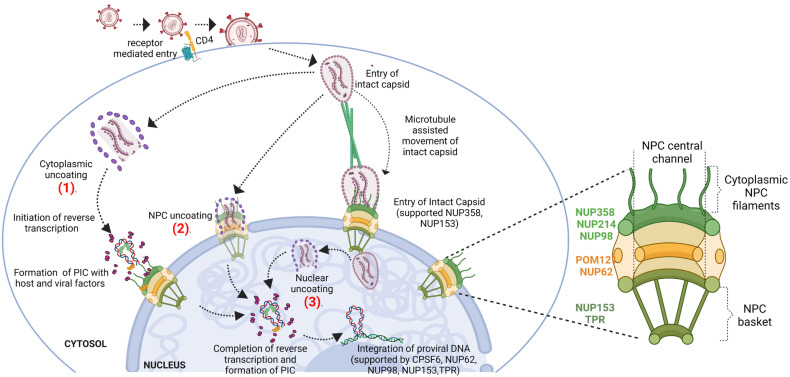
Schematic representation of the models depicting the import of HIV-1 in a host cell. HIV-1 enters the host cell by fusion with the host membrane releasing its intact capsid into the cytoplasm. With the help of the microtubular network, the intact capsid is transported to the cytoplasmic periphery of the nucleus. The uncoating of the capsid occurs either in the cytoplasm (1) or at the NPC (2) or in the nucleus (3). As detailed in the text, the interaction of capsid with NUP153, NUP358, and other host proteins favors nuclear import. The disassembly of the capsid, reverse transcription, and PIC formation occur in the nucleus. The proviral DNA is integrated into the host genome supported by CPSF6, NUP62, NUP98, and NUP153. (1), (2) and (3) refer to the Schemes 1, 2 and 3 described in the text. Inset: The nucleoporins of NPC associated with the import of capsid and integration of the HIV-1 genome are listed.

The experimental evidence in the field supports that capsid core may enter the nucleus in multiple ways; while a few may enter intact, others are partially broken up in the cytoplasm or near the NPCs at the cytoplasmic side of the nuclear membrane before ingressing into the nucleus, as schematically shown in Figure 1.

Early investigations were suggestive of the capsid disassembly (uncoating) in the cytoplasm [17,18,19] or at the nuclear envelope [20,21] before the import of viral DNA into the nucleus. The most prominent basis for this postulation was the relatively larger size of the conical capsid core (broad end; 60 nm) that surpasses the diameter of the NPC channel (previously described as 40 nm) for it to be transported. While the model of cytoplasmic uncoating was based on both fluorescence microscopy and a biochemical assay that leverages the restriction of HIV-1 infection by TRIM-Cyp (cyclosporine A (CsA)-washout assay), the reports that proposed nuclear membrane-associated uncoating of the capsid largely relied only on the fluorescence microscopy data. In microscopy studies, fluorescence tags were introduced into the assembling virions to determine the status of the capsid during the early steps of HIV-1 replication. These tags were genetically fused directly to the viral components such as Gag as a fluid phase marker, Integrase (IN), and Vpr, or indirectly to the capsid-associated host factors such as cyclophilin A (CYPA) to track the virion particles used for the infection [17,22]. The loss of capsid-associated fluorescent signal upon cellular entry could therefore be marked as the uncoating of the incoming capsid. The analysis of fluorescently-labelled single viral particles post-entry indicated that the majority of the capsid cores lost the fluorescent signal immediately after viral membrane fusion, while a few retained the signal for some time, losing it eventually in the cytosol, although productive infection was observed. These imaging experiments were taken as indirect clues of the capsid uncoating to be a cytosolic event [17,22]. The observation that the concomitant loss of both CYPA and CA signals in the cytosol as well as at the nuclear membrane suggested that an uncoating event might be occurring in the cytosol and at the nuclear membrane [22]. The studies utilizing biochemical CsA-washout assay, which is based on the ablation of restriction to the HIV-1 infection imposed by TRIM-Cyp by the addition of CsA and the determination of the percentage of HIV-1 infected cells that become insensitive to TRIM-Cyp (relative half-life of uncoating), also support the model that CA is gradually lost from the capsid core with a half-life of less than 1 h in the cytosol. While these assays laid a critical foundation for investigating capsid uncoating, they also opened up multiple interpretations such as, the CsA might interfere with interactions between capsid and CYPA-domain containing host factors including CYPA that otherwise might maintain the integrity of the capsid [18,19,23].

It was, however, shown later that the subviral complexes formed upon uncoating in the cytosol were subjected to proteasomal degradation and could not generate productive infection, indicating that cytosolic uncoating may not be favorable for HIV-1 [21]. In addition, later studies showed that the capsid cores could dock at NPC and undergo disassembly before entering into the nucleus as subviral complexes [20,21]. Nevertheless, to support the model of nuclear uncoating, recent culminating evidence from the studies of fluorescence microscopy, wherein CA was directly fused to green fluorescence protein, suggests that the intact or nearly intact capsids enter and disassemble in the nucleus for successful integration and productive infection [24,25,26]. Studies also further indicate that the capsid, while maintaining its integrity, is imported through the NPC, translocated to the sites of integration, and uncoats before integration into the genome [24,25]. In line with this evidence, Zila et al., by using combined correlative light and electron microscopy, showed that the diameter of the NPC is sufficiently dilated for the import of intact cone-shaped capsid into the nucleus of T cells [26]. While this experimental evidence clearly pointed to the possibilities of various modes of capsid uncoating, many of them employed virus preparation wherein, labelled viral proteins were trans-complemented with wild-type proteins. This may lead to differential labelling of individual virus particles, confounding the microscopy data interpretation, necessitating the use of less invasive labelling strategies to study the early stages of HIV-1 infection, including uncoating. Recently, Schifferdecker et al. established a method, wherein CA is directly labelled with fluorescent dye through genetic code expansion and click chemistry as a less invasive strategy and showed that largely intact capsids enter the nucleus indicating uncoating is a nuclear event that precedes integration [27]. Apart from the general conception that the size of the capsid limits access to the nucleus, the differences in the methods, the timing of viral tracking, and the number of particles analyzed in the studies might explain the discrepancy in their conclusion from the new paradigm of nuclear uncoating. Based on these pieces of evidence, one may infer that the models of uncoating described above may not be mutually exclusive and vary depending on the cell type and conditions used in the infection. These models of uncoating are depicted as three schemes (1–3) in Figure 1. It is, therefore, possible to conceive of the following events during the capsid journey toward the nucleus via cytoplasm.

##### Movement towards the Nucleus

Post entry into the cytosol, while some capsid cores encasing HIV-1 RTC/PIC may undergo disassembly in the cytosol (Figure 1, Scheme 1), some are transported to the cytoplasmic side of the nuclear envelope via CA-microtubule interactions [28,29,30]. Dynein-associated adaptor protein, bicaudal D2 (BICD2), facilitates CA association with microtubules, resulting in the movement toward the nucleus [29,31,32]. Complementing the same, the ultrastructural studies by Zila et al. showed that about majority of the capsids proximal to NPC were associated with microtubules, and the average distance of capsids with microtubules was 19 ± 12 nm, which is in agreement with the distance between microtubule and dynein/kinesin-1 as reported earlier [14,33,34]. It has been observed that during HIV-1 infection, the Kinesin-1 motor KIF5B induces the relocalization of NUP358 into the cytoplasm [35]. The relocalization allows the interaction between NUP358 and incoming capsid cores for the import of HIV-1. Further, the critical residues (N74 and P90) on the CA are shown to be required for the interaction between NUP358 and CA, and therefore the mutation of these residues (N74D and P90A) induced the accumulation of capsid cores near the cytoplasmic face of the nuclear envelope indicating that NUP358 promotes capsid import into the nucleus [35]. HIV-1, like other viruses, also uses plus end-directed motor protein (kinesin) and its adaptor protein FEZ1 (fasciculation and elongation factor zeta 1) involved in the anterograde transport for net-inward movement of capsid core toward the nucleus [29]. The authors of this study proposed a model wherein the opposing motors contribute to the bidirectional movement of HIV-1 to accomplish the net retrograde movement of HIV-1.

##### Ingress into the Nucleus through NPC

Two persuasive events can be envisaged for capsid upon nearing the nuclear membrane: a) the docking of capsid cores onto the NPC promotes the accelerated loss of capsid monomers from the intact capsid, and these partially disassembled subviral complexes enter into the nucleus (Figure 1, Scheme 2) [20,21]. (b) Physically compatible diameter of the central channel of NPC allows the intact capsid to penetrate the nucleus without hampering the typical cone shape of HIV-1 capsids (Figure 1, Scheme 3) (although morphological alterations of capsid core were observed once inside the nucleus) [26]. An interesting observation by Zila et al. was that, unlike what was conventionally reported, the central channel of human NPC (in SupT1 cells) was sufficiently dilated with an average diameter of about 64 nm, which is well above the dimension of the broad end of the conical shaped capsid core by about 4 to 9 nm, therefore allowing geometrically feasible entry into the nucleus [26]. Intriguingly, this property of dilation of NPC was observed in both infected and uninfected SupT1 cells. The moving capsid cores encounter a high concentration of NUP FG repeats in the central channel.

##### Release from NPC into the Nucleoplasm

NUP153 and CPSF6 (cleavage and polyadenylation specificity factor 6) are encountered by the capsids when they leave the NPC central channel toward the nuclear side. Upon traversing to the nuclear basket present on the nuclear face of NPC, the sub-viral complex interacts with NUP153 through a conserved hydrophobic pocket on CA hexamers to complete its translocation to the nucleus and release PIC [23,36,37]. NUP153 is a nuclear basket protein that, although is positioned to the nuclear face of NPC, has a 200 nm long C-terminal carrying an unstructured FG domain, can reach toward the cytoplasmic face of NPC, where it interacts with PIC-associated CA, integrase (IN), and Vpr of the partially opened capsid core [37,38,39,40]. The nuclear protein CPSF6, which is an RNA processing protein, binds to the same site on CA as NUP153. It is presumed that CPSF6 replaces NUP153 at the common binding site on CA, that subsequently releases the capsid containing PIC into the nucleus [26,37]. However, a mechanistic model for the movement of partially opened or intact capsids and their eventual release into the nucleus is yet to be determined. One may wonder if CA binding factors, such as CPSF6, from the inside of the nucleus, generates an inward force, or there is a gradient of (FG) binding sites to the capsid core inside the channel of NPC, or it could be a result of the variable CA concentrations in cone-shaped capsid core at opposite poles of NPCs.

A comprehensive pictorial representation of HIV-1 capsid core translocation from the cytoplasm to the nucleus and its integration is shown in Figure 1. The host and viral proteins associated with the ingress of the HIV-1 capsid core into the nucleus are listed in Table 1.

#### 2.1.2. Factors Associated with Capsid Uncoating

Though the capsid core protects the genome from being detected by the cellular innate sensors [53,54,55], the same becomes the target for the cellular restriction factors including TRIM5α (tripartite motif 5α) and MX2 (myxovirus resistance 2) that bind and inactivate the capsid [56,57]. Yet, a recent study by Yoh et al. showed that intact capsid is coated by an adaptor protein PQBP1 (polyglutamine binding protein1) which initiates cGAS-dependent innate immune response [58]. The details regarding the sensing of HIV-1 viral components as a whole by the host cell and the associated evading strategies by HIV-1 are beyond the scope of this review, and hence, readers may refer to the recent reviews [17,21,24,55,59]. We here discuss the factors associated with capsid uncoating.

CYPA, a cytosolic peptidyl-prolyl isomerase, is known to bind to the proline-rich region, namely CYPA binding loop, in the N-terminal domain of HIV-1 CA [60]. In this binding loop, the amino acids, glycine 89 and proline 90 were shown to be crucial for the CA interaction and their changes (G89V and P90A) led to decreased HIV-1 infection [61,62,63,64]. Consequently, CYPA binding to CA was shown to either stabilize or destabilize the HIV-1 capsid in a cell type-dependent manner, indicating its role in the modulation of HIV-1 capsid disassembly [64]. The interaction of nuclear transport receptor transportin 1 (TRN-1) with CA has been shown to promote both the uncoating and import of the HIV-1 genome. This interaction has been mapped to the NLS located in the CYPA binding loop of CA [65]. Another nuclear transport receptor, transportin SR2 (TRN-SR2)/transportin 3 (TNPO3), has been shown to promote the uncoating of HIV-1 cores [66]. However, the detailed mechanism of TNPO3 action during HIV-1 infection remains to be established, given the fact that its depletion has indirect consequences (discussed below). NUP358 contains a cyclophilin domain by which it binds to the CA at the same site where CYPA binds [67]. Though the experimental evidence pointed to the peptidyl-propyl isomerase activity of NUP358, it is still unclear if this enzyme activity leads to the structural changes in the capsid, possibly causing uncoating [68,69]. While it may be speculated that opposing stabilizing and destabilizing forces possibly decide the fate of HIV-1 core uncoating, conclusive in vivo experiments to validate the factors involved in uncoating remain to be explored. Whereas we focus here on the involvement of nuclear trafficking proteins. A step-by-step narration of the uncoating of HIV-1 core that discusses all cellular factors involved is elaborated in some recent reviews [70,71].

#### 2.1.3. Reverse Transcription and Integration of Viral Genome

##### Reverse-Transcription

For productive infection by HIV-1, the (+) strand RNA genome must be reverse-transcribed into double-stranded DNA by the reverse transcriptase (RT) enzyme and integrated into the host genome by the integrase. Briefly, the RT needs the 3’ OH of a primer to start polymerization, similar to all other DNA polymerases. A cellular tRNA already present within retroviral particles serves as the primer for (−) strand synthesis and anneals to the primer binding site (PBS) in the 5’ region of the genome to initiate reverse transcription. The process of reverse transcription of viral RNA has been extensively reviewed and may be referred to in [72,73] and references therein. Though the cellular location of the beginning of reverse transcription remains to be elucidated, the mounting evidence suggests the completion of reverse transcription in the nucleus [25,74]. Further, several reports demonstrate a coordinated relationship between the uncoating and reverse transcription [18,75,76,77,78,79]. Rankovic et al. using time-lapse atomic force microscopy showed that the early stage of reverse transcription increases the pressure inside the capsid core that triggers the capsid disassembly [80]. In agreement with this data, it was shown that the first strand transfer synthesis, the initial stage of reverse transcription, is required for the capsid to disassemble but not the late products of the reverse transcription [81]. In parallel, Francis et al. utilizing fluorescence microscopy for single viral particle tracking observed the gradual loss of CYPA-DsRed from capsid upon initiation of reverse transcription, loss of this fluorescence indicating the uncoating [22]. While these studies strengthen the paradigm that reverse transcription promotes the uncoating, experimental evidence shows that the alterations in capsid stability, by the introduction of point mutations in CA, impair the process of reverse transcription, substantiating a coordinated relationship between these processes [75,82].

Further, it is proposed that the viral capsid can act as a container to maintain an effective concentration of RT molecules and possibly other components required for the reverse transcription, and given the fact that RT frequently dissociates from the template and reverse transcription requires two template switching events, intact capsid may therefore ensure to hold the RT molecules near the sites of reverse transcription [79]. To the best of our knowledge, nuclear transport proteins have not been directly linked to the reverse transcription of the viral genome. However, host factors, some of which are either positive or negative regulators, have a significant regulatory role in the process of viral genome reverse transcription [72].

##### Integration

The linear double-stranded viral DNA (vDNA), the product of reverse transcription, complexes with integrase and host factors to form an ill-defined sub-viral complex called the preintegration complex (PIC), and the vDNA associated with this complex must be integrated into the chromatin of the infected cells by viral IN in a two-step process (3′ processing and strand transfer). The viral genetic material becomes a part of the cellular genome once it has been transformed into DNA and integrated, rendering the cell permanently infected. Therefore, as long as the infected cell continues to divide, the provirus is copied and faithfully inherited [83].

Though several host factors associated with PIC have been identified, their functional role in HIV-1 integration remains elusive [84]. Many of the transport receptors including KPNA2/KPNB1, KPNA4, TRN-1, and TNPO3 [85] and NUPs including NUP62 [44] and NUP153 [38] have been shown to interact with IN, while their molecular function in the integration is yet to be elucidated. The depletion of NUP62 has been shown to reduce its abundance with host chromatin and cause a decrease in the viral integrations, suggesting its direct role in the integration that depends on the IN interaction [44]. Though NUP153 can interact with both IN and CA, the relevance of these interactions either in the import or integration needs to be functionally elucidated [38,40,42]. In addition, NUP98 is also shown to be involved in the integration of viral DNA but the mechanism behind this function is still unknown [40]. Given the fact that certain NUPs including NUP98 and NUP153 are mobile and move on and off the NPC [86,87], it is plausible that their dynamic nature of interactions with viral determinants might perform required functions at required places. For instance, NUP153 can promote both PIC import by interacting with CA at NPC and its integration into the genome in the nucleoplasm by interacting with IN.

Another important aspect of productive HIV-1 infection is the trafficking of PIC to the integration sites and, thus, its selection in the genome. Accumulating evidence indicates that HIV-1 favors gene-dense regions that are transcriptionally active, particularly genomic regions that are close to the speckle-associated domains (SPADs) while disfavoring the heterochromatin regions such as lamina-associated domains (LADs) for its integration [43,88,89,90]. Several host factors have been shown to play an important role in selecting HIV-1 integration sites in the genome. Among these, LEDGF/p75 (lens epithelium-derived growth factor) and CPSF6 have been extensively studied for their role in targeting the viral genome in gene-rich regions [89,90,91,92]. Though these proteins, through their differential interacting viral determinants (LEDGF/p75 with IN; CPSF6 with CA), help viral integrations into relatively gene-dense regions, the zonal locations of integration in the nucleus were shown to vary for each of these proteins. While LEDGF/p75 promotes integration in the nuclear periphery, CPSF6 helps integration into the nuclear interior regions that are proximal to SPADs [43,90,93]. Further studies are required to clarify these differential interactions and their effects on the integration site selection. Interestingly, it has been recently observed that though initially the transcriptionally active proviruses were seen in the nuclear periphery (near the nuclear envelope), after a few cell divisions, these proviruses were observed throughout the nucleus, indicating that the nuclear distribution of transcriptionally active proviruses was dynamic, and the distance of the location of HIV-1 proviruses from nuclear envelope does not correlate with its transcriptional activity [94].

In parallel, studies have shown that NUPs, namely NUP358 and NUP153, and TNPO3 are involved in the integration of viral DNA into gene-dense regions [95,96]. Depletion of NUP358 and TNPO3 caused alterations in the integration site selection (from gene-rich regions to gene-sparse regions), and this effect was mapped to depend on the HIV-1 Gag [96]). It is suggested that the reduction of HIV-1 infection upon TNOP3 depletion seemed to be an indirect consequence of the cytoplasmic accumulation of CPSF6, which is restrictive for infection, and therefore the role of TNPO3 in integration site selection remains to be answered [97]. Like NUP358, the nuclear basket protein NUP153 was shown to interact, although at a different region, with CA to promote integration into gene-rich regions [40,95]. Integration site analysis of HIV-1 with N57 mutations that disrupt the interaction between capsid and NUP153 showed reduced integration into transcriptionally active genes similar to HIV-1 in the cells devoid of CPSF6, indicating that the integration site selection depends on several factors in the infected cells [42]. Although other NUPs, including NUP214, NUP98, and NUP62 were shown to interact with CA, their participation in the integration site selection remains to be elucidated [41,44].

### 2.2. Translocation of Viral Factors from the Nucleus to the Cytoplasm

The transcriptional regulation of the integrated HIV-1 proviral DNA revealed a complex interplay of host factors and the long terminal repeat (LTR) promoter of HIV-1. Some of the nucleoporins, such as NUP153, NUP98, NUP62, and TPR (translocated promoter region) were shown to interact with HIV-1 LTR with the silencing of NUP153 and TPR negatively affecting the viral transcription [43]. The connection between nucleoporins and viral DNA transcription, however, is still under investigation.

**Figure 2:**
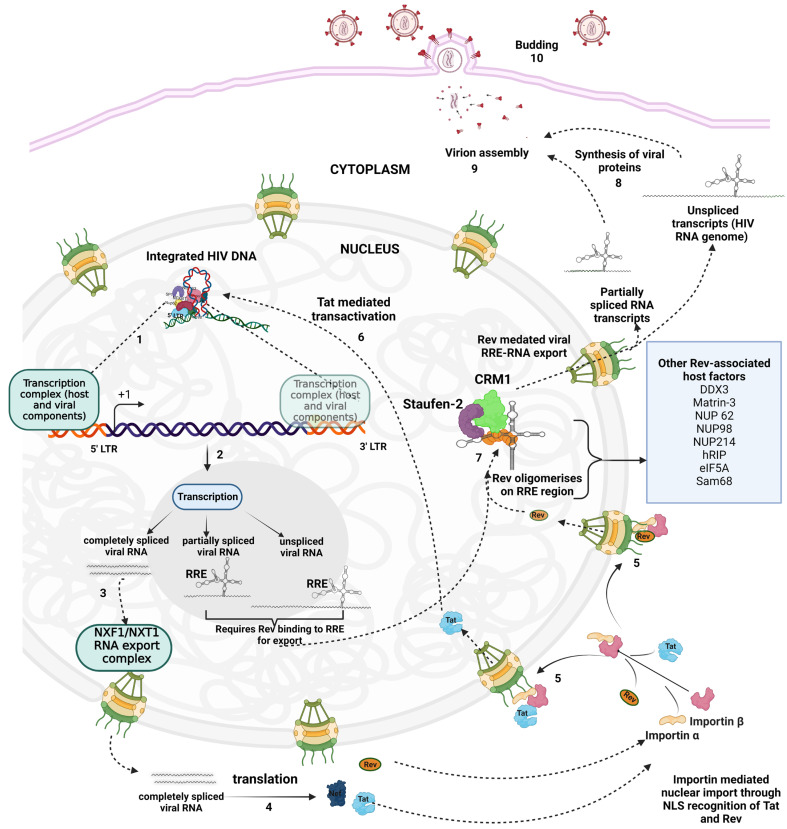
Schematic representation of events depicting post-integration events during HIV-1 infection, representing some of the crosstalk of viral components with nuclear trafficking machinery. The integrated viral DNA (1) at the sites of euchromatic regions is transcribed by the host transcription machinery; some of the experimentally demonstrated proviral DNA binding proteins are listed in the Table 1. Transcription leads to the generation of three kinds of viral mRNA, unspliced, partially spliced, and completely spliced (2). The completely spliced viral transcripts are exported by the host NXT1/NXF1 system (3). The viral proteins Rev, Tat, and Nef are generated upon translation of the completely spliced RNA (4). Rev and Tat are imported into the nucleus through importin α/β complex by their NLS signal sequence (5). Tat translocates into the nucleus and causes transactivation of viral LTR promoter (6), Rev binds to Rev responsive elements (RRE) present in the partially spliced and unspliced viral RNAs and interacts with several host RNA export factors (listed in the box) and mediates transport of RRE containing viral RNA into the cytoplasm (7). The partially spliced and unspliced RNAs translate different structural and regulatory proteins (8) and are assembled into the viral core along with two copies of the RNA genome (unspliced viral RNA) (9). The assembled virions bud out of the cell (10).

Transcription from the viral LTR produces full-length viral mRNA, which upon splicing generates partially spliced or completely spliced RNA. The completely spliced (2kb) viral mRNA encodes three early proteins Tat, Rev, and Nef [98]. Nef, an important early factor that helps in establishing HIV-1 infection, once synthesized in the cytoplasm, is not known to enter the nucleus. It downregulates cell-surface proteins, such as MHC I, and CD4, facilitating evasion of elimination by cytotoxic T lymphocytes (CTLs), and preventing superinfection respectively [99,100]. It also counteracts the antiviral SERINC proteins and downregulates many other cell-surface proteins [101,102]. The two other early regulatory proteins, Tat and Rev, enter the nucleus. Tat acts as a trans-activator, while Rev, shuttles across the nuclear membrane to carry the cargo of partially- and unspliced viral RNAs. The viral mRNA for the accessory and structural proteins (Gag, Env, Pol) is translated in the next phase once the partially spliced (4.5kb) and unspliced RNA (9.5kb) reach the cytoplasm marking the late phase of infection [98]. We discuss the impact of proteins involved in the nuclear transport of HIV-1 Tat and Rev in the following sections. Figure 2 presents a comprehensive illustration of the nucleocytoplasmic export of viral RNAs and proteins. The host and viral proteins associated with the export of HIV-1 RNAs and proteins are listed in Table 2.

#### 2.2.1. Transportation of Tat from the Cytoplasm to the Nucleus, and Viral Transcription

Tat is synthesized from completely spliced viral mRNA that is possibly exported by the conventional heterodimeric transport receptors Tap-p15 (NXF1-NXT1) in mammalian hosts [123]. Tat is a trans activator generated during infection, without which the bulk of viral transcriptions terminate prematurely [124]. Tat binds to the TAR element in the viral RNA [125,126]. After its translation in the cytoplasm, the trans-activator protein must find its way back into the nucleus. Smith et al. were the first to structurally establish that the eight amino acids (GRKKRRQR) within the Tat NLS region form a direct and stable contact with the nuclear import receptor importin-α [127]. Proteomic and mass spectrometry-based approaches identified several nucleoporins (NUP85, NUP88, NUP93, NUP98, NUP153, NUP205, NUP358) and nuclear transport factors (RAN, RANGAP1, XPO1, KPNA2, KPNB1) to be associated with Tat. While their role in Tat transportation to the nucleus is yet to be deciphered, it is also suggested that they may play a role in controlling the provirus’s transcriptional state [128].

In the absence of Tat, transcription initiation occurs at LTR but prematurely ends at TAR, resulting in short, dead-end RNA. The positive transcription elongation factor b (pTEFb) binds to 6 nucleotide loop in the TAR hairpin in Tat dependent manner [129] enabling the cyclin-dependent kinase 9 (CDK9), a component of pTEFb, to phosphorylate the hydroxyl groups in C-terminal tail of RNA polymerase II enhancing its processivity. Though Tat is known to function as a transcriptional activator, it can also regulate splicing which is dependent on co-transcriptional splicing activators- CA150 and Tat-SF1 (Tat stimulatory factor 1) [130]. Tat tightly regulates the splicing process, because the production of spliced and unspliced RNAs must be balanced for optimal virus replication. Tat also promotes the use of splice sites in mRNAs specific for Rev or Env/Nef [131]. Tat has many additional activities in HIV-1 infection, such as; it induces apoptosis, releases neurotransmitters, causes oxidative stress, and causes inflammation [132].

#### 2.2.2. Transportation of Rev from the Cytoplasm to the Nucleus

The N-terminal region of Rev contains an arginine-rich domain and the NLS for Rev to enter the nucleus [133]. Henderson et al. showed that the Rev NLS mediates a direct and highly specific interaction with importin-β [134]. Importin-β binds to the GDP-bound form of RAN (Ran-GDP) to target Rev into the nucleus. In the nucleus, the conversion from Ran-GDP to Ran-GTP dissociates Rev from importin-β and allows Rev’s binding to the RRE in viral pre-mRNAs [135]. Importin-β does not bind to the Rev-RNA complex, ensuring that Rev is imported only after the release of the RNA cargo [134]. Besides importin-β, transport receptors such as importin-5, importin-7, and transportin also interact with Rev to promote its transport inside the nucleus. It was found that the arginine-rich NLS of Rev is required for the interaction with all these importins [135].

#### 2.2.3. Transportation of Unspliced and Partially Spliced Viral RNAs from the Nucleus to the Cytoplasm by HIV-1 Rev and Associated Nucleocytoplasmic Trafficking Machinery

HIV-1 Rev is the viral factor associated with the export of partially spliced and unspliced viral RNA from the nucleus to the cytoplasm, with the help of a plethora of host transport accessory proteins. Transport by Rev allows the translation of structural and accessory components of the virion, resulting in a high rate of virus production [136]. It was observed that Rev deficient virus is not able to form new virus particles due to negligible export of unspliced and partially spliced viral mRNA shedding light on the significance of Rev in the viral life cycle. Delayed expression or differential compartmentalization of Rev can affect the level of HIV-1 infection in a host cell [137].

Recent experiments also point to an unclear role of NUP62 in viral RNA export, where Monette et al. showed that NUP62 knockdown caused the retention of viral RNA in the nucleus like what is observed in Rev deficient conditions. NUP62 was found to be packaged into new virions, and colocalization analyses by them have shown NUP62 to localize with Rev, viral RNA, and Gag, thus supporting a non-NPC role for NUP62 [52]. However, owing to a lack of convincing experimental evidence, it is difficult to assess the role of NUP62 in HIV-1 RNA export from the nucleus to the cytoplasm.

Rev mediates the transport of unspliced and partially spliced viral mRNA, which encodes structural and accessory proteins, by binding to Rev responsive element (RRE) present in these RNAs [137,138,139]. HIV Rev binding element (RBE), which is a part of RRE, is located in the 2nd intron downstream of the *env* gene [140]. Rev binding to HIV-1 RBE (within RRE) causes the import complex to dis-assemble eventually exposing Rev nuclear export signal (NES) that interacts with the export receptor, chromosome region maintenance 1 (CRM-1), also known as exportin 1 [141,142]. Rev dimerizes on RRE that positions its C-terminal domains (CTDs) spatially for the efficient recruitment of CRM1 [115]. The process requires the GTP-bound form of the Ran GTPase. Briefly, in the nucleoplasm, guanine nucleotide exchange factor (GEF) converts Ran-GDP to Ran-GTP that associates with CRM1. The RanGTP-CRM1 complex binds to the exposed NES of RRE-bound Rev. The RRE-Rev-CRM1-RanGTP complex is then exported out via NPC through CRM-1 interactions with NUPs, such as NUP98, NUP214 [51,143]. The cargo of RRE-Rev is released in the cytoplasm with the conversion of Ran-GTP to Ran-GDP by Ran-GAP (Ran-GTPase activating protein). The unequal distribution of Ran-GEF and Ran-GAP across the nuclear membrane warrants a continual RAN-GTP/GDP gradient to enable CRM-1 recycling and sustained Rev/RRE export [141,144]. In the cytoplasm, Rev dissociates from RRE-RNAs exposing its NLS to interact with importins for its way back to the nucleus [134,145].

Besides RanGTP-CRM1, several factors act as accessory molecules influencing cellular distribution Rev and Rev-mediated RNA export activity and have been briefed here. Phosphofurin acid cluster sorting protein 1 (PACS1) was identified as a CRM1-associated protein in the human nuclear complexosome [146]. Liu et al. observed that overexpression of PACS1 increases the level of unspliced viral RNA [146]. PACS1 acts as a Rev cofactor involved in viral replication.

The cellular cofactor known as human Rev-interacting protein (hRIP) is necessary for Rev-dependent viral RNA export across the nuclear membrane [118,147]. Rev-directed RNAs mislocalize and abnormally accumulate near the nuclear periphery, in the absence of functional hRIP [118]. Phenylalanine-glycine (XXFG) repeat sequences are abundant in hRIP and are similar to the GLFG and XFXFG repeats found in the majority of mammalian nuclear pore proteins [148]. Based on its structure and intracellular distribution, hRIP is likely to play a role in the trafficking or localization of RNA, probably by interaction with CRM1 and mRNA nuclear export factor 3 (NXF3) [148]. At the cytoplasmic site of the nuclear pore complex, it is suggested that hRIP can facilitate the disassembly of Rev-directed RNA [147].

Src-associated protein, Sam68 (KHDRBS1) directly contributes to the nuclear export of HIV-1 partially spliced or unspliced RNA through its interaction with the HIV-1 Rev protein [149]. In effect, it was initially observed to be capable of substituting and working in concert with the HIV-1 Rev protein and later shown to form a protein-protein complex with Rev in vivo, via Rev NES and the region between amino acid residues 321 and 410 [121]. Sam68 binds RRE, most likely via the UAAA sequence found within stem I of RRE [121]. It can work with RNA helicase A and TAP to expedite the export of RRE-containing RNA [150]. Rev functional deficit in astrocytes is correlated with a reduced level of constitutive Sam68, and overexpressing Sam68 in these cells corrects the Rev defect [151].

Staufen homolog 2 (Staufen 2) is involved in the transport and/or localization of mRNAs to various subcellular organelles and/or compartments [152]. Human Staufen 2 has multiple splice variants and forms ribonucleoprotein complexes [153]. Studies have established that human Staufen 2 interacts with Rev and positively regulates the RRE-containing RNA export activity of Rev to promote progeny virus synthesis [117,154,155].

A highly conserved inner nuclear matrix protein, Matrin3 (MATR3) is attached to the inner nuclear membrane and forms a skeletal nuclear framework. RRE-containing HIV-1 transcripts are stabilized and exported from the nucleus to the cytoplasm when Matrin3 interacts with Rev [156]. It contains two RNA recognition motifs (RRMs) within amino acids 399 to 567, as well as a bipartite NLS in amino acids 586 to 612. Matrin3 binds to RNA through these RRMs, the deletion of which restricts the protein to the nucleus [156]. The cellular protein, polypyrimidine tract binding protein associated binding factor (PSF) also interacted with Matrin 3 where their interaction with Rev occurs only via RNA. With these, it is speculated that RRE containing viral incompletely spliced RNA are marked to export via MATR3/Rev by PSF [157].

HIV-1 recruits several DEAD-box family of proteins, which facilitate the optimum metabolism of the viral RNAs to ensure viral gene expression [158]. Helicases such as DDX1, DDX3, DDX5, and DHX9 assist in transcription, nuclear RNA export, and translation initiation of HIV-1 proteins [159,160]. Unspliced viral RNA export is facilitated by DDX3 through CRM-1, depletion of which decreased Rev-mediated export and virion synthesis [119,120]. Yeast and mammalian two-hybrid assays identified DDX1 as a cofactor of HIV-1 Rev [161]. siRNA-mediated knockdown of DDX1 in Cos-1 and HEK293 cells, altered the subcellular localization of Rev, making it abundant in the cytoplasm, which correlated with decreased viral production [162]. However, overexpression of DDX1 enhanced viral titers. Mechanistically, the chaperone activity of DDX1 by facilitating the conformational change in RRE promoted the oligomerization of Rev on RRE [163]. RNA-mediated Rev-DDX5 interaction also promotes Rev RNA export activity [164]. DDX21, also known to enhance Rev activity, localizes with Rev in the nucleolar and peri-nucleolar regions [165]. Several other Dead box helicase proteins colocalize with Rev and interact with Tat, pointing to their role in the export of viral mRNA; however further studies are required to elucidate their defined roles [162].

The Rev-dependent nuclear export of HIV-1 RNA has been linked to eukaryotic initiation factor 5a (eIF5a) [166], which is known to interact with CRM1 and associated with intra-nuclear filaments, thereby participating in nuclear transport machinery [167]. It was discovered to be a cofactor for the human T-cell leukemia virus type I Rex protein and the HIV type 1 Rev protein. eIF5A regulates the movement of viral mRNAs from the nucleus to the cytoplasm using ribosomal protein L5 which is the central component of the 5S rRNA export system [168].

While we limited our description to a few factors supported by conclusive experimental evidence, the recent report by Knoener et al. [169] on viral splice variant protein interactomes has expanded the list of host factors that may also participate in viral RNA transport. Further investigations are required to associate these factors with Rev-dependent or Rev-independent mechanisms of viral RNA nuclear export, which may lead to a new area in HIV-1 biology. Rev, being a multi-faceted protein, interacts with several host cell components at various stages of the HIV-1 life cycle, discussion on which is out of the scope of this review. For a comprehensive account of Rev-associated host factors, the cited review [115] and the references therein may be referred.

Finally, with the viral RNA exported to the cytoplasm, viral proteins are synthesized and appropriately packed with the unspliced HIV-1 RNA as the genome for emerging virus particles, thus completing the infection cycle.

## 3. Concluding Remarks

With the emergence of drug-resistant HIV-1 strains, it is imperative to look into new host pathways that can be targeted, and nucleocytoplasmic trafficking of viral factors has been one such event, which has attracted attention for decades to regulate HIV-1 propagation. Though it was presumed that the researchers had deciphered the mechanism of HIV-1 nuclear import-export strategies, the virus has surprised the scientific community with its versatility in hijacking a battery of host factors, so much, so that targeting the import-export pathways of the virus has not been very successful. While it is obvious that without the aid of these host factors, the movement of HIV-1 into and outside the nucleus will be unfeasible, the primary theme that emerges from the above discussion is the utilization of redundant factors and pathways for nucleocytoplasmic transport of HIV-1 factors, makes it highly challenging to conceive these interactions for host-directed therapy. Besides the transport of viral factors, the nucleocytoplasmic shuttling also influences other host cellular processes associated with the success of HIV infection, such as cell cycle regulation mediated by GNL3L [170].

Even though all the proteins that we discussed above belonged to the category of positive regulators of HIV-1, one of the ILF-3 genes, which codes for nuclear factor 90 (NF90), is a negative regulator of HIV-1 Tat and Rev. NF90 belongs to the family of proteins with double-stranded RNA binding domain (DRBD). The C-terminal variant, NF90ctv, is distinct from other proteins encoded by ILF-3 in that its C-terminal 67 amino acids is Arg-Gly-poor (RG-) and is substituted by acidic amino acid residues, whereas the corresponding C-terminal region is Arg-Gly-rich (RG+) in all the other proteins of this family. The RG domain of NF90ctv is possibly involved in blocking Rev-mediated viral RNA export. NF90 can also directly interact with RRE-RNA structure, indicating a competition between Rev and NF90ctv, which ultimately affects RNA export to the cytoplasm [116]. The TAR RNA binding ability of NF90ctv inhibits the Tat-mediated transactivation of HIV-1 LTR to attenuate HIV-1 replication and contribute to antiviral response [171]. The list of nuclear transport-associated proteins as potential negative regulators is minimal. A comparative account of nucleocytoplasmic trafficking factors in permissive and reservoir cells or cells, that warrant strict regulations with limited HIV-1 propagation, will help identify host factors with anti-HIV-1 properties. Investigating these antiviral factors may further our understanding of the human host’s defense mechanisms and anti-retroviral strategies.

Further, it will be interesting to investigate if the differential expression of positive regulators correlates with disease progression in a population and/or influence R5-X4 conversions within a single host. In addition, HIV-1 can also serve to unveil new mechanisms underlying the physiological transport of macromolecules between the cytoplasm and the nucleus in eukaryotic hosts.

## Figures and Tables

**Table 1:** List of nucleoporins and nuclear pore complex (NPC) associated proteins that participate in nucleocytoplasmic trafficking of HIV-1 factors.

S. no.		Factor	Event	Role in HIV-1 Replication	Reference
1	During the import of Viral RNA/Cargo, and proviral DNA integration	NUP358/RanBP2	Docking/Import	Mediates docking of HIV-1 capsid to the cytoplasmic face of NPC and subsequent import	[35,41]
2		NUP153	Import	Binds HIV-1 CA for translocation through the nuclear pore using FG repeats, and involved in the integration site selection, and is shown to bind to the proviral DNA	[40,41,42,43]
3		NUP62	Integration	By interacting with HIV-1 integrase, it promotes the integration of proviral DNA and is also shown to bind to the proviral DNA	[43,44]
4		NUP98	Integration	It is involved in the HIV-1 integration into the host chromatin, and is also shown to bind to the proviral DNA	[40,41,43]
6		NUP88	Import	Interacts with the capsid and thus involved in the import	[45]
7		TRN-SR2	Import and integration	HIV-1 IN interacts with HEAT repeats of TRN-SR2	[46]
8		Pom121	Import	Mediates HIV-1 PIC nuclear import by binding to KPNB1	[47]
9		Importin α3	Import	Imports HIV-1 PIC by interacting with HIV-1 IN	[48]
10	Export of Viral RNA/Cargo post-integration	CRM-1	Export of viral RNA	Binds to Rev NES and facilitates its export	[49]
11		NUP98	Export of viral RNA	Involved in the CRM1 mediated Rev export	[50]
12		NUP214	Export of viral RNA	Required for CRM1- dependent nuclear protein export	[51]
13		NUP62	Export of viral RNA	Promotes the export of vRNA	[52]

**Table 2:** List of additional host proteins (other than nucleoporins and NPC-associated proteins) and viral proteins involved in the nucleocytoplasmic trafficking of HIV-1. Some of the listed host proteins act as negative regulators of HIV-1 trafficking (*).

S No		Factor	Role in HIV-1 Replication	References
1	Cytoplasm to nucleus	CPSF6	Interacts with HIV-1 CA for nuclear entry, integration targeting	[103,104]
2		LEDGF/p75	Directs HIV PIC to transcriptionally active sites and IN cofactor	[105,106,107]
3		Cyclophilin A	Interacts with HIV-1 CA and prevents restriction of HIV-1 by TRIM5α	[108]
4		MX2 *	Interacts with HIV-1 CA and reduces levels of uncoating and integrated proviral DNA	[109,110]
5		Kinesin-1	Composed of KIF5A, KIF5B, and KIF5C promote uncoating using FEZ1 and NUP358	[111]
6		TRIM5α *	Binds HIV-1 CA followed by degradation of the viral core by ubiquitin-dependent & independent pathways	[112]
7		HIV-1-Capsid	Import of RNA by protecting from restriction factors	[113]
8		HIV-1 Integrase	Catalyzes the viral DNA integration into the host genome	[114]
9	Nucleus to cytoplasm	HIV-1 Rev	Facilitates unspliced/partially spliced RRE containing RNA transport	[115]
10		Nuclear Factor 90 *	Blocks Rev mediated export function	[116]
11		Staufen-2	Complexes with CRM1-Rev to enhance Rev-mediated RNA export	[117]
12		hRIP	Cellular cofactor of Rev	[118]
13		DDX3	Facilitates viral RNA Export	[119,120]
14		Sam68	Interacts with HIV-1 Rev protein and exports viral RNA	[121]
15		eIF5A	Binds to HIV-1 Rev activation domain	[122]

## Data Availability

Not applicable.
